# Risk Factors for Unexpected Admission Following Lumbar Spine Laminectomy: A National Database Study

**DOI:** 10.7759/cureus.55507

**Published:** 2024-03-04

**Authors:** John M Tarazi, Petros Koutsogiannis, Emma K Humphrey, Nabil Z Khan, Michael Katsigiorgis, Gus Katsigiorgis, Randy M Cohn

**Affiliations:** 1 Department of Orthopedic Surgery, Donald and Barbara Zucker School of Medicine at Hofstra/Northwell, Hempstead, USA; 2 Department of Orthopedic Surgery, Northwell Health-Huntington Hospital, Huntington, USA; 3 Department of Orthopedic Surgery, Ohio University Heritage College of Osteopathic Medicine, Warrensville Heights, USA; 4 Department of Orthopedic Surgery, New York Institute of Technology College of Osteopathic Medicine, Old Westbury, USA; 5 Department of Orthopedic Surgery, Northwell Health-Long Island Jewish Valley Stream, Valley Stream, USA

**Keywords:** orthopedic spine surgery, lumbar spine, spine, rate of readmission, laminectomy

## Abstract

Introduction

Laminectomy is one of the most common orthopedic spine surgeries performed in the United States. Compared to other spine operations such as fusions, laminectomies in isolation are of lower morbidity. However, complications may arise that result in readmission to an inpatient healthcare facility. The purpose of this study is to identify the demographics and risk factors associated with unplanned 30-day readmission following a laminectomy.

Methods

The American College of Surgeons National Surgical Quality Improvement Program (ACS-NSQIP) database was queried for patients who underwent a laminectomy procedure from 2015 to 2019 using CPT code 63030. This query yielded 61,708 cases. Demographic, lifestyle, comorbidity, and peri-operative factors were recorded. Independent samples Student’s *t*-tests, chi-squared, and, where appropriate, Fisher’s exact tests were used in univariate analyses to identify demographic, lifestyle, and peri-operative variables related to 30-day readmission following a laminectomy procedure. Multivariate logistic regression modeling was subsequently performed. Odds ratios (ORs) with 95% confidence intervals (CIs) were calculated and reported.

Results

Of the 61,708 patients included in our sample, 2,359 were readmitted within 30 days of surgery, corresponding to a readmission rate of 3.82%. Results of the univariate analysis revealed statistically significant relationships between readmission status and the following patient variables: patient age, sex, BMI, ASA classification, race, bleeding disorder, chronic obstructive pulmonary disease (COPD), diabetes, hypertension, congestive heart failure (CHF), chronic steroid use, total operative time, and tobacco use (p < 0.05). Multivariate logistic regression modeling confirmed that the following patient variables were associated with statistically significantly increased odds of readmission: age greater than 65 (p < 0.05), female sex (p = 0.013), bleeding disorder (p = 0.011), diabetes (p = 0.006), current smoker (p = 0.010), COPD (p < 0.001), steroid use (p = 0.006), ASA Class II or above (p < 0.05), and total operative time (p < 0.001).

Conclusion

Unplanned 30-day readmission after laminectomy is infrequent. However, increasing age, female sex, steroid use, current smokers, bleeding disorders, diabetes, COPD, CHF, a higher ASA classification, and longer operative times are independent risk factors for readmission following laminectomy.

## Introduction

Spinal laminectomies are a common surgical procedure within orthopedic surgery designed to decompress the spinal cord or underlying nerves through the removal of the lamina [1]. Patients suffering from conditions such as lumbar spinal stenosis, a degenerative condition of the spinal discs and other surrounding structures, often undergo this procedure for pain relief and restoration of physical movement and function [1-4]. Although regarded as a better pain-relieving option than non-surgical treatments, patients may develop post-surgical complications that require inpatient hospital readmission [1,5-8].

Patients who undergo laminectomy procedures report significantly better post-operative Oswestry Disability Index (ODI) scores compared to other common spinal operations and are associated with reduced leg pain and need for reoperation [5,9]. Specifically, patients who undergo lumbar laminectomy procedures have reported better quality of life and lower reoperation rates compared to patients undergoing spinal fusion procedures [10]. Although recent studies have demonstrated that laminectomy procedures are associated with lower morbidity compared to other procedures, risk factors for readmission have not been thoroughly investigated.

There are certain patient risk factors that may contribute to higher chances of readmission following surgical laminectomies and it is imperative to evaluate potential predictors of patient readmission. Compared to other spine operations such as fusions, laminectomies in isolation are of lower morbidity. However, complications may arise that result in readmission to an inpatient healthcare facility. Therefore, the purpose of this study is to identify the demographics and risk factors associated with unplanned 30-day readmission following a laminectomy.

## Materials and methods

Database

This study utilized the American College of Surgeons National Surgical Quality Improvement Program (ACS-NSQIP) database. Data was collected by trained clinical reviewers from over 700 participating hospitals and include patient demographics, comorbidities, surgery in Current Procedural Terminology (CPT) codes, diagnoses in International Classification of Disease 9th and 10th (ICD-9, ICD-10, respectively) revision codes, and 30-day post-operative surgical outcomes.

Patient population

The ACS-NSQIP database was queried for patients who underwent a laminectomy procedure from 2015 to 2019 using CPT code 63030, yielding 61,708 cases.

Variables collected

The following demographic, lifestyle, and comorbidity variables were recorded: age, sex, body mass index (BMI), American Society of Anesthesiologists (ASA) classification, bleeding disorders, chronic obstructive pulmonary disease (COPD), diabetes mellitus, hypertension, congestive heart failure, chronic steroid use, and tobacco use. Peri-operative variables that were collected included anesthesia type (general versus regional), and total operative time. The primary outcome of 30-day readmission was defined as unplanned hospital readmission related to the principal procedure.

Statistical analyses

All data were analyzed using the Statistical Package for the Social Sciences (SPSS) version 23.0 (IBM Corp., Armonk, NY). The criterion for statistical significance was set at α = 0.05. Independent samples Student’s t-tests, chi-squared, and, where appropriate, Fisher’s exact tests were used in univariate analyses to identify demographic, lifestyle, comorbidity, and peri-operative variables related to 30-day readmission following laminectomy. Multivariate logistic regression modeling was subsequently performed. Odds ratios (ORs) with 95% confidence intervals (CIs) were calculated and reported.

## Results

Of the 61,708 patients included in our sample, 2,359 were readmitted within the 30-day post-operative period, corresponding to a readmission rate of 3.8%. Demographic, lifestyle, comorbidity, and peri-operative factors are presented in Table 1. The mean age of the non-admitted cohort was 51.54 ± 15.5 versus the mean age of the admitted cohort of 55.75 ± 16.3 (p < 0.001). Moreover, the mean BMI of the non-admitted cohort was 30.27 ± 6.5 versus the admitted cohort of 31.17 ± 6.9 (p < 0.001). The mean operative time of the non-admitted cohort was 91.5 ± 55 (p < 0.001) versus that of the admitted cohort which was 106.67 ± 66.5 (p < 0.001).

**Table 1:** The relationship between demographic, lifestyle, comorbidity, and peri-operative factors and readmission following laminectomy *American Society of Anesthesiologists

	Not Admitted		Admitted		P-value
	(N = 59,349)		(N = 2,359)		
	N	Percent (%)	N	Percent (%)	
Outcome					
Age					< 0.001
<65	45,230	96.7	1,538	3.3	
65-79	12,086	94.8	668	5.2	
80+	2,010	92.9	153	7.1	
Sex					< 0.001
Male	33,375	96.5	1,226	3.5	
Female	25,974	95.8	1,133	4.2	
Body Mass Index, kg/m^2^					< 0.001
Underweight	378	95.7	17	4.3	
Normal weight	11,357	96.8	370	3.2	
Overweight	20,533	96.4	766	3.6	
Obese, Class I	14,727	96.1	604	3.9	
Obese, Class II	7,108	95.8	310	4.2	
Obese, Class III	4,593	94.7	256	5.3	
ASA* Classification					< 0.001
Class I	6,101	98.1	116	1.9	
Class II	34,352	97	1,061	3	
Class III	18,053	94.3	1,090	5.7	
Class IV	782	89.9	88	10.1	
Class V	3	100	0	0	
Bleeding Disorder	378	90.2	41	9.8	< 0.001
Chronic Obstructive Pulmonary Disease	1,401	90.5	147	9.5	< 0.001
Diabetes	8,079	94.2	501	5.8	< 0.001
Hypertension	22,742	95	1,190	5	< 0.001
Congestive Heart Failure	94	84.7	17	15.3	< 0.001
Steroid Use	2,003	93.1	149	6.9	< 0.001
Tobacco Use	13,128	95.7	583	4.3	0.003
Anesthesia Type					0.874
General	59,003	96.2	2,346	3.8	
Regional	288	96	12	4	
Total Operative Time	59,339	91.5 ± 54.95	2,359	106.67 ± 66.46	< 0.001

Results of univariate analysis revealed statistically significant relationships between readmission status and the following patient variables: Patient Age, t(61,683) = 13.47, p < 0.001; Sex, χ2 (1, 61,708) = 16.75, p < 0.001; BMI, t(61,017) = 15.2, p < 0.001; ASA, χ2 (4, 61,646 = 406.96, p < 0.001; Bleeding Disorder, χ2 (1, 41,744) = 36.67, p < 0.001; COPD, χ2 (1, 61,708) = 139, p < 0.001; Diabetes, χ2 (1, 61,708) = 110.2, p < 0.001; Hypertension, χ2 (1, 61,708) = 140.52, p < 0.001; CHF, χ2 (1, 61,708) = 39.95, p < 0.001; Steroid Use, χ2 (1, 61,708) = 58.32, p < 0.001; Tobacco Use, χ2 (1, 61,708) = 8.83, p = 0.003; and total operative time, t(61,696) = 13.04, p < 0.001. Anesthesia type was not significantly associated with readmission.

Multivariate logistic regression modeling confirmed that the following patient variables were associated with statistically significantly increased odds of readmission (see Table 2): Age, 65-79, p = 0.003, OR 1.22, 95% CI [1.07, 139]; Age, 80+, p < 0.001, OR 1.58, 95% CI [1.25, 2]; Sex, p = 0.013, OR 1.15, 95% CI [1.03, 1.28]; Bleeding Disorder, p = 0.011, OR 1.61, 95% CI [1.12, 2.33]; Diabetes, p = 0.006, OR 1.21, 95% CI [1.06, 1.40]; Tobacco Use, p = 0.01, OR 1.19, 95% OR [1.04, 1.35]; COPD, p < 0.001, OR 2.00, 95% CI [1.59, 2.51]; CHF, p < 0.05, OR 1.94, 95% CI [1.00, 3.77]; Steroid Use, p = 0.006, OR 1.38, 95% CI [1.10, 1.73]; ASA Class II, p = 0.002, OR 1.55, 95% CI [1.18, 2.04]; ASA Class III, p < 0.001, OR 2.40, 95% CI [1.81, 3.18]; ASA Class IV, p < 0.001, OR 3.60, 95% CI [2.38, 5.43]; and Total Operative Time, p < 0.001, OR 1.10, 95% CI [1.102, 1.104]. The relationship between BMI, Hypertension, Anesthesia Type, and readmission did not achieve statistical significance in the multivariate model.

**Table 2:** Multivariate analysis related to outcomes of interest *American Society of Anesthesiologists

Outcome	OR	95% CI	P-value
Age (reference < 65)			
65-79	1.22	(1.07, 1.39)	0.003
80+	1.58	(1.25, 2.00)	< 0.001
Female Sex (reference = male)	1.15	(1.03, 1.28)	0.013
Bleeding Disorder	1.61	(1.12, 2.33)	0.011
Diabetes	1.21	(1.06, 1.40)	0.006
Tobacco Use	1.19	(1.04, 1.35)	0.01
Chronic Obstructive Pulmonary Disease	2	(1.59, 2.51)	< 0.001
Congestive Heart Failure	1.94	(1.00, 3.77)	< 0.050
Steroid Use	1.38	(1.10, 1.73)	0.006
ASA* (reference = Class I)			
Class II	1.55	(1.18, 2.04)	0.002
Class III	2.4	(1.81, 3.18)	< 0.001
Class IV	3.6	(2.38, 5.43)	< 0.001
Total Operative Time	1.1	(1.102, 1.104)	< 0.001

## Discussion

Spinal laminectomies are performed with successful patient outcomes and reduced morbidity in comparison to other spinal operations; however, patient readmissions have been reported and are associated with multiple risk factors. The purpose of this study was to determine various risk factors with post-laminectomy readmission. Of the variables studied, the patient risk factors that were found to be statistically significant were patient age, sex, BMI, ASA classification, race, bleeding disorder, COPD, diabetes, hypertension, CHF, chronic steroid use, operative time, and tobacco use. Through multivariate regression, any patients who were above the age of 65, female, underwent longer procedure times within the OR, had existing CHF, COPD, bleeding disorders, steroid use, or smoking history were at increased risk for 30-day unplanned readmission following the laminectomy. Additionally, patients classified as overweight or obese (I-III) were also at increased risk for readmission.

Alongside other authors, the authors of this study have demonstrated an association between set variables and readmission, such as increased age. Although our study examined more variables than a 2014 study conducted by Basques et al., there were similar relationships produced with certain patient variables, such as BMI and age [11]. Among a sample size of 2,358 patients, the results of Basques et al. demonstrated that increasing age (greater than 65 years) was strongly associated with readmission [11]. While examining readmissions within 30 days after surgery, Basques et al. similarly determined that both increased BMI (obese classes II and III especially) and patient steroid use placed patients at higher risk for readmission, which agrees with both of our findings on these same associations [11]. Fujihara et al. interestingly discovered that patients with comorbidities such as diabetes were at a significantly (p = 0.001) higher risk for post-laminectomy fractures around the isthmus region which resulted in readmission [12]. It is unclear whether or not the correction for these fractures resulted in readmission within 30 days post-operatively for their smaller sample size (n = 92). Still, nonetheless, their study parallels our findings in that diabetes places post-laminectomy patients at greater risk for readmission due to post-operative complications [12].

Longer operation times have resulted in an increased risk for readmission within 30 days post-laminectomy. Additionally, as discovered by Darveau et al., these increased operative times resulted in an increased risk for further intervention, such as the necessity for a blood transfusion [13]. In an attempt to associate the need for blood transfusions with post-laminectomy and fusion surgeries, Darveau et al. determined that patients who were in surgery for a median of 267 minutes (compared to 176) put them at significantly higher risk (p < 0.001) for receiving a blood transfusion [13]. Furthermore, receiving a transfusion nearly doubled their risk for 30-day readmission, from 4.9% to 9.6% [13]. While the risk of readmission associated with transfusion was not evaluated in this study, longer operative times were shown to predispose patients to unplanned readmission [13]. The inclusion of spinal fusions could worsen reported patient outcomes, as previous papers mentioned such as one from Mohanty et al. found that spinal fusions are associated with worsened morbidity when compared to laminectomies [10]. However, in a significant contrast to our study, Elsamadicy et al. determined that when comparing laminectomy and non-laminectomy cohorts of patients undergoing spinal fusion, patients who underwent laminectomies had significantly higher (p < 0.001) incidences of post-operative critical care admissions and higher incidences of mental health status changes (p = 0.05) within 30 days post-operatively [14]. This contrasts our findings, as our study determined that laminectomies in isolation are associated with lower morbidity. However, this difference could be due in part that fusions are also part of these patients’ surgical procedures, and that patients are receiving a spinal fusion regardless, either with or without a laminectomy [14]. Elsamadicy et al. also discovered that there was no difference between the two cohorts in 30-day readmission risks, which further contrasts our results that patient risk factors significantly influence readmission rates following a laminectomy in general [14].

Overall, our study evaluated a multitude of patient risk factors that significantly affect the risk of unplanned readmission to a healthcare facility within 30 days of undergoing a laminectomy procedure. Although spinal laminectomies are considered routine and are of lower patient morbidity, pre-existing patient factors can lead to post-operative complications. Through multivariate regression analyses, unplanned readmission significantly increases due to a variety of patient demographics and risk factors, such as increased BMI, age of over 65 years, female sex, pre-operative diabetes, COPD, CHF, race, hypertension, smoking status, ASA classification, steroid use, and bleeding disorders. Over the years, additional studies may be performed to further explore these risk factors and uncover the exact impact on readmission. With such findings, patient-surgeon treatment plans and pre-operative risk assessments can be modified to include the awareness of how the patient’s risk factors may influence their post-operative outcomes. Although not emphasized within this particular study, this approach will not only benefit the patient but also may reduce costs with regards to anesthetics from reduced operation times and demand for medications and resources post-operatively. Hospital systems will also benefit from recognizing and implementing patient risk factors into surgical plans, as fewer complications and better patient satisfaction may improve the hospital’s reputation and efficiency with patient care and turnover. With these results, it is important to mention that complications may arise without any of these risk factors due to the nature of unpredictability within the OR, but nonetheless, it is a good foundation for future readmission reduction within orthopedics. Orthopedic surgeons must always keep their patient’s best interests in mind, especially when taking into consideration the reality of individual post-operative outcomes that are rational for that patient. This paper improves the awareness of post-operative readmission for the orthopedic society as a whole based on the findings of patient risk factors influencing their odds of being readmitted within 30 days following orthopedic surgery, specifically spinal laminectomies.

This study is not without limitations. The study contains a few limitations aside from its retrospective nature. The others of this study did not differentiate between patients who had a laminectomy at one versus multiple levels. Additionally, there are limitations when using the NSQIP database. The NSQIP database queries CPT codes to identify patients. This allows for the potential of miscoding leading to incomplete patient capture. In addition, post-operative data is only collected for 30 days. Complications can often occur outside of the 30-day period, including DVT, PE, infections, reoperation, readmission, and more. Therefore, this study limits the ability to evaluate complications that occur outside of this post-operative time and underestimates the overall complication rate. The database is reported by participating hospitals and thus may not be generalizable to all patient populations. It may not include free-standing surgery centers. This data could then potentially be excluded from the data cohort. This would likely result in an overall lower readmission rate.

## Conclusions

Although 30-day readmission following laminectomy procedures is infrequent, a better understanding of these risk factors can aid physicians to better educate their patients, as well as limit admissions from reoccurring. In our study, independent risk factors for readmission included: increasing age, female sex, steroid use, current smokers, bleeding disorders, diabetes, COPD, CHF, a higher ASA classification, and longer operative times. Higher odds of readmission were seen with patients who are greater than 65 years old, females, current smokers, use steroids, have bleeding disorders, diabetes, COPD, ASA Class II+, and longer operative times. Surgeons should consider alternative treatments to limit the risk of readmission after treatment of the laminectomy procedure when possible.

## References

[1] Wei FL, Zhou CP, Liu R (2021). Management for lumbar spinal stenosis: a network meta-analysis and systematic review. Int J Surg.

[2] Soffin EM, Beckman JD, Beathe JC, Girardi FP, Liguori GA, Liu J (2020). Trends in ambulatory laminectomy in the USA and key factors associated with successful same-day discharge: a retrospective cohort study. HSS J.

[3] Lurie JD, Tosteson TD, Tosteson A, Abdu WA, Zhao W, Morgan TS, Weinstein JN (2015). Long-term outcomes of lumbar spinal stenosis: eight-year results of the Spine Patient Outcomes Research Trial (SPORT). Spine (Phila Pa 1976).

[4] Weinstein JN, Lurie JD, Tosteson TD (2008). Surgical versus nonoperative treatment for lumbar disc herniation: four-year results for the Spine Patient Outcomes Research Trial (SPORT). Spine (Phila Pa 1976).

[5] Epstein NE (2018). Lower complication and reoperation rates for laminectomy rather than MI TLIF/other fusions for degenerative lumbar disease/spondylolisthesis: a review. Surg Neurol Int.

[6] Schöller K, Alimi M, Cong GT, Christos P, Härtl R (2017). Lumbar spinal stenosis associated with degenerative lumbar spondylolisthesis: A systematic review and meta-analysis of secondary fusion rates following open vs minimally invasive decompression. Neurosurgery.

[7] Lad SP, Babu R, Baker AA (2013). Complications, reoperation rates, and health-care cost following surgical treatment of lumbar spondylolisthesis. J Bone Joint Surg Am.

[8] Patil CG, Sarmiento JM, Ugiliweneza B (2014). Interspinous device versus laminectomy for lumbar spinal stenosis: a comparative effectiveness study. Spine J.

[9] Richter A, Koutsoumbelis SA, Essig DA (2014). Comparison between laminectomy and interspinous devices for treatment of lumbar spinal stenosis. Spine J.

[10] Mohanty S, Kadiyala M, Barchick S (2022). Patient-reported and clinical outcomes after lumbar laminectomy and fusion versus laminectomy alone in patients with lumbar spondylolisthesis and harmonious sagittal spinopelvic alignment. Spine J.

[11] Basques BA, Varthi AG, Golinvaux NS, Bohl DD, Grauer JN (2014). Patient characteristics associated with increased postoperative length of stay and readmission after elective laminectomy for lumbar spinal stenosis. Spine (Phila Pa 1976).

[12] Fujihara R, Komatsubara S, Arima N, Yamamoto T (2020). Scoliosis, diabetes mellitus and total laminectomy at the 4th lumbar vertebra are independent risk factors for post-laminectomy fracture around the isthmus. Neurochirurgie.

[13] Darveau SC, Pertsch NJ, Toms SA, Weil RJ (2021). Short term outcomes associated with patients requiring blood transfusion following elective laminectomy and fusion for lumbar stenosis: a propensity-matched analysis. J Clin Neurosci.

[14] Elsamadicy AA, Adogwa O, Warwick H (2017). Increased 30-day complication rates associated with laminectomy in 874 adult patients with spinal deformity undergoing elective spinal fusion: a single institutional study. World Neurosurg.

